# Zinc Deficiency and the Recurrence of *Clostridium difficile* Infection after Fecal Microbiota Transplant: A Retrospective Cohort Study

**DOI:** 10.1155/2018/9682975

**Published:** 2018-10-10

**Authors:** Blake A. Niccum, Daniel J. Stein, Brian W. Behm, R. Ann Hays

**Affiliations:** ^1^Department of Internal Medicine, Massachusetts General Hospital, Boston, MA, USA; ^2^Department of Internal Medicine, Division of Gastroenterology, Beth Israel Deaconess Medical Center, Boston, MA, USA; ^3^Department of Internal Medicine, Division of Gastroenterology and Hepatology, University of Virginia Health System, Charlottesville, VA, USA

## Abstract

**Background:**

Fecal microbiota transplant (FMT) is an effective therapy for recurrent *Clostridium difficile* infection (CDI). However, in 12% of patients treated with FMT, CDI recurs within one month. Zinc deficiency predicts increased diarrheal frequency in malnourished children, but little is known about its association with FMT outcome. We hypothesized that zinc levels were an independent predictor of CDI recurrence after FMT.

**Methods:**

We performed a retrospective cohort study of 80 patients (mean age, 66; 59 women) receiving FMT for CDI from 9/2013–9/2016 at a tertiary care center. Zinc levels were measured within 90 days before FMT. The primary outcome was CDI recurrence within 90 days after FMT. We controlled for risk factors for FMT failure using Cox regression. We also analyzed the effect of zinc supplementation in individuals with deficiency.

**Results:**

Forty-nine subjects had a normal zinc level, and 31 had a low level (<0.66 *µ*g/mL). CDI recurred in 3/49 (6%) patients with normal zinc and 5/31 (16%) patients with low zinc (HR = 11.327, 95% CI = 2.162–59.336, *p*=0.004). Among low zinc subjects, 2 of 25 (8%) that received zinc supplements and 3 of 6 (50%) that did not receive zinc supplements had recurrence of CDI (HR = 0.102, 95% CI = 0.015–0.704, *p*=0.021).

**Conclusion:**

Zinc deficiency was associated with increased CDI recurrence after FMT. Among zinc-deficient patients, supplementation was associated with reduced recurrence. Further study is needed to determine whether zinc deficiency represents a pathophysiologic mechanism and target for therapy.

## 1. Introduction

*Clostridium difficile* infection (CDI) is a leading cause of healthcare-associated illness. The incidence of CDI in hospitalized adults in the US nearly doubled from 2001 to 2010, increasing from 4.5/1,000 to 8.2/1,000 [1]. In 2011, approximately 453,000 CDIs occurred in the US and were associated with an estimated 29,000 deaths [2].

Initial CDI classically occurs following use of antibiotics and results from the inadvertent eradication of gut microbes less resilient than spore-forming *C. difficile* [3]. As many of these microbes and their metabolites serve to suppress *C. difficile*, their removal from the microbiome facilitates the disproportionate and deleterious expansion of toxin-forming *C. difficile* strains, leading to CDI [3].

Treatment of CDI with antibiotics (metronidazole, vancomycin, or fidaxomicin) is frequently complicated by recurrence, further raising disease burden. This is hypothesized to be the result of persistent alteration of the colonic microbiome [4]. Approximately 20% of patients treated for initial CDI experience recurrence [5], and 45–65% of those with history of at least one recurrence will have more recurrences [6, 7].

Fecal microbiota transplant (FMT) is currently the most effective therapy for patients with recurrent CDI [8]. Approximately 85% of patients treated with FMT for recurrent CDI experience resolution of symptoms [8]. However, up to 12% of patients experience recurrence of CDI within one month after FMT [9].

Established risk factors for CDI recurrence after FMT include inpatient status at time of FMT, infusion of donor stool into the upper gastrointestinal (GI) tract, use of FMT for severe or severe-complicated CDI, higher number of previous CDI-related hospitalizations, and a diagnosis of inflammatory bowel disease (IBD) [9–11]. However, these risk factors do not fully explain variation in outcomes, and few offer targets for intervention.

Zinc, an essential trace element in the human body, regulates enzymatic activity and gene expression to facilitate broad physiologic processes such as immune function, protein synthesis, and wound healing [12]. Zinc is obtained from dietary intake and is primarily lost via the GI tract in feces as sloughed zinc-laden intestinal cells, unabsorbed dietary zinc, and unabsorbed endogenous zinc from intestinal secretions [12]. The prevalence of zinc deficiency is estimated at 31% globally and 4–7% in the United States [13]. Risk factors for zinc deficiency include inadequate zinc intake, impaired intestinal absorption (as in disorders such as inflammatory bowel disease), and excessive intestinal losses (seen in diarrheal illness) [12, 14].

Zinc deficiency is an established risk factor for diarrheal illness, predicting both diarrheal frequency and severity in malnourished pediatric populations [15]. Among IBD patients, depletion of zinc has been associated with increased disease-related morbidity [16]. Thus, zinc deficiency both results from and perpetuates GI illness. In children, zinc supplementation reduces diarrheal incidence when used prophylactically and decreases diarrheal duration, severity, and mortality when used during active diarrheal illness [17, 18]. Although the precise antidiarrheal mechanisms of zinc remain unclear, the benefit of zinc in the management of diarrhea is evident; the WHO recommends zinc supplementation in the treatment of acute diarrheal illness [19].

Despite evidence for zinc as an antidiarrheal agent and the likelihood that CDI places patients at risk for zinc deficiency, zinc has never been studied in humans in the context of either CDI or FMT. In this study, we evaluated whether low serum zinc was associated with increased recurrence of CDI within 90 days after FMT. Among subjects with zinc deficiency, we investigated whether zinc supplementation could decrease recurrence. We hypothesized that zinc deficiency would be associated with increased CDI recurrence, and that receipt of zinc supplements by those with low serum zinc would be associated with decreased recurrence.

## 2. Materials and Methods

### 2.1. Study Cohort

This retrospective cohort study included all adults (≥18 years) who received FMT via outpatient colonoscopy for recurrent CDI between September 2013 and September 2016 at the University of Virginia Health System who had a serum zinc value measured within 90 days before FMT. Measurement of serum zinc was included in the routine clinical evaluation of patients with recurrent CDI as many of these patients had a history of chronic diarrhea, IBD, and/or elderly age, all risk factors for zinc deficiency [12]. This study was approved by the University of Virginia Institutional Review Board, and all data were collected via examination of electronic medical records.

All CDI episodes occurring prior to FMT were recorded. CDI was defined by the presence of diarrhea (≥3 or more watery stools in 24 hours) and positive *C. difficile* stool test (PCR or toxin ELISA) [20]. Recurrent CDI was defined by the presence of diarrhea and positive *C. difficile* stool test occurring ≥ 14 days after previously diagnosed and appropriately treated CDI. Episodes of recurrent diarrhea empirically treated as CDI recurrence without stool testing were recorded as “undocumented” CDI episodes. Severe CDI was defined as CDI with serum albumin <3 g/dL and the presence of either abdominal tenderness or white blood cell (WBC) count ≥15,000 cells/mm^3^ [20]. Severe-complicated CDI was defined as CDI with any of the following: admission to the intensive care unit for CDI, hypotension with or without the required use of vasopressors, fever ≥ 38.5 degrees Celsius, presence of ileus, mental status changes, WBC ≥ 35,000 or <2,000 cells/mm^3^, serum lactate > 2.2 mmol/L, or any evidence of end-organ failure [20].

Recurrence of CDI after FMT, the primary outcome, was defined as the re-emergence of diarrhea and positive *C. difficile* stool test within 90 days following FMT. Follow-up after FMT was obtained via examination of medical record notes detailing clinic visits and routine post-FMT telephone calls.

In addition to basic demographic information, data pertaining to medical history and established risk factors for FMT failure were collected, including the number of prior CDI-related hospitalizations and the presence of IBD (ulcerative colitis, Crohn's disease, or indeterminate colitis) [9, 11]. To control for other risk factors for FMT failure, no patients were included that received FMT while hospitalized, via an upper GI route, or within 14 days following severe or severe-complicated CDI [9, 10]. Patients were considered immunocompromised if they had any of the following: HIV infection (any CD4 count), AIDS-defining diagnosis or CD4 < 200/mm^3^, inherited or primary immune disorders, or immunosuppression from a medical condition or medication used within the preceding three months (including but not limited to antineoplastic agents, monoclonal antibodies to B and T cells, antitumor necrosis factor agents, glucocorticoid equivalents to prednisone ≥ 20 mg/day, antimetabolites (azathioprine, 6-mercaptopurine, and methotrexate), calcineurin inhibitors (tacrolimus and cyclosporine), and mycophenolate mofetil) [9]. Albumin, WBC count, and C-reactive protein (CRP) were recorded at the time of zinc measurement.

### 2.2. Recurrence of CDI after FMT

Individuals were categorized as zinc replete or deficient based on our institutional cut-off for normal serum zinc of 0.66 *µ*g/mL. Baseline characteristics were compared using either *t*-test or chi-square (or Fisher's exact) test as appropriate. Follow-up interval was recorded at the last available clinic or telephone follow-up. Time of interest was determined to be 90 days based on our clinic follow-up patterns and unlikely relationship between FMT and CDI recurrences at greater than 90 days. All individuals were categorized as either “recurrence of CDI post-FMT” or “censored” (in the case of loss to follow-up before 90 days, death before 90 days, or 90 days of follow-up without CDI recurrence). Given variable follow-up time, FMT time-to-recurrence outcomes were first compared using Kaplan–Meier curves with the Wilcoxon test. Given the retrospective nature of the data, we then conducted our primary analysis with Cox regression.

Based on risk factors for FMT failure or CDI in general, we entered the following variables into the Cox regression model: number of prior CDI-related hospitalizations, presence of IBD, immunocompromised status, Charlson Comorbidity Index, and age [9, 11, 21]. We also entered gender and receipt of zinc supplementation based on bivariate calculations. To select the final model, backward selection with *p* < 0.1 was used as the staying criterion. Proportionality for the Cox regression was assessed using visual comparison with Kaplan–Meier curves for categorical variables and the Kolmogorov-type supremum test for continuous variables. For all testing, significance was set as *p* < 0.05.

Subjects with low zinc levels were categorized based on receipt of zinc supplementation starting within 90 days before FMT to up to 14 days after FMT. Individuals experiencing recurrence of CDI after FMT prior to starting zinc supplementation were categorized as unsupplemented. FMT outcomes were analyzed identically to the complete cohort via use of both Kaplan–Meier curves and Cox regression. The same factors were included in this second regression except for zinc supplementation.

All statistical analysis was conducted via SAS version 9.3.

## 3. Results

### 3.1. Study Cohort

A total of 127 patients were treated with FMT for CDI at our institution between September 2013 and September 2016; 95 patients had zinc levels measured within 90 days pre-FMT, and 80 met study criteria (Table 1). The population mean age was 66 and included 59 (74%) women and 21 (26%) men. All subjects received FMT via outpatient colonoscopy for recurrent CDI: 74 (93%) had history of at least three documented CDI episodes, five (6%) had history of two documented episodes plus at least one undocumented episode and one episode requiring hospitalization, and one (1%) had history of one documented episode plus two undocumented episodes.

Before FMT, 61% (49 of 80) of subjects had normal and 39% (31 of 80) of subjects had low serum zinc levels as defined by our institutional range for normal serum zinc (0.66–1.10 *µ*g/mL) (Table 1). Subjects with normal zinc had mean zinc of 0.77 ± 0.09 *µ*g/mL (mean ± SD), and subjects with low zinc had mean zinc of 0.52 ± 0.08 *µ*g/mL.

### 3.2. Recurrence of CDI after FMT

**Figure 1:**
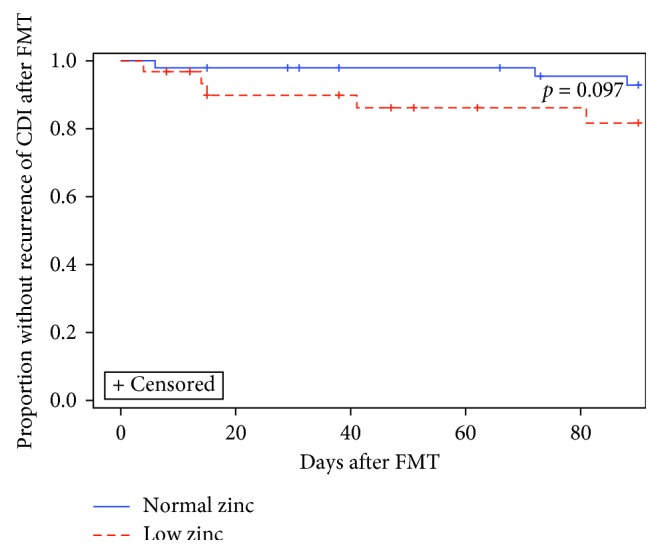
Recurrence of CDI after FMT: normal vs. low zinc. When analyzed without controlling for risk factors for FMT failure, low zinc was not associated with increased *Clostridium difficile* infection (CDI) recurrence, with 6% (3/49) of normal zinc and 16% (5/31) of low zinc subjects experiencing recurrence within 90 days after FMT (*p*=0.097, Wilcoxon).

Overall, 10% (8 of 80) of patients experienced CDI recurrence within 90 days after FMT (Table 2). Time from FMT until CDI recurrence was a median of 28 days and ranged from 4 to 88 days. In the unadjusted Kaplan–Meier analysis, low zinc was not associated with increased CDI recurrence, with 6% (3 of 49) of normal zinc and 16% (5 of 31) of low zinc subjects experiencing CDI within 90 days after FMT (*p*=0.097) (Table 2; Figure 1). However, in our primary analysis controlling for potential confounding variables using Cox regression, the overall rate of CDI recurrence was higher in the zinc-deficient group compared with zinc-replete individuals (HR = 11.327, 95% CI = 2.162–59.336, *p*=0.004). Increased Charlson Comorbidity Index was also associated with increased CDI recurrence (HR = 1.441, 95% CI = 1.087–1.911, *p*=0.011), while receipt of zinc supplementation was associated with decreased recurrence (HR = 0.119, 95% CI = 0.019–0.738, *p*=0.022). All other variables were removed as nonsignificant predictors (*p* > 0.1) (Table S1).

Among subjects with low zinc, 81% (25 of 31) received zinc supplementation (Table 3).

Subjects were treated by multiple providers, and supplementation was prescribed at physician discretion. When specified, zinc supplementation was typically prescribed at doses of 25–50 mg nightly (22 of 23; 96%) for 1-2 months (19 of 20; 95%).

**Figure 2:**
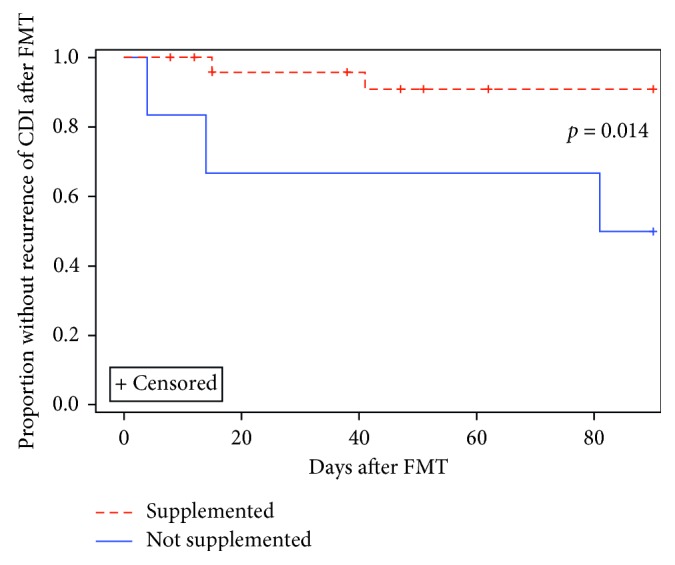
Recurrence of CDI after FMT among low zinc subjects. Among low zinc subjects, repletion of zinc was associated with reduced *Clostridium difficile* infection (CDI) recurrence, with 8% (2/25) of supplemented and 50% (3/6) of unsupplemented subjects experiencing recurrence within 90 days after FMT (*p*=0.014, Wilcoxon).

Repletion of zinc was associated with reduced CDI recurrence, with 8% (2 of 25) of supplemented and 50% (3 of 6) of unsupplemented subjects experiencing CDI within 90 days after FMT (*p*=0.014, Wilcoxon) (Table 2; Figure 2). Using Cox regression, receipt of zinc supplementation was associated with decreased CDI recurrence (HR = 0.102, 95% CI = 0.015–0.704, *p*=0.021). Increased Charlson Comorbidity Index was associated with increased recurrence (HR = 1.521, 95% CI = 1.015–2.279, *p*=0.011). All other variables were removed as nonsignificant predictors (*p* > 0.1) (Table S2).

With respect to bivariate analysis, zinc deficiency correlated with the presence of IBD: 4% (2 of 49) of patients with normal zinc had IBD compared to 32% (10 of 32) of patients with low zinc (*p* < 0.001). Importantly, however, the rate of CDI recurrence within 90 days after FMT was not statistically significantly different between patients with and without IBD (2/12 patients with IBD vs. 6/68 patients without IBD, *p*=0.344). Zinc deficiency also correlated with male gender (*p* < 0.001), low albumin (<3.5 g/dL) (*p*=0.001), and elevated CRP (≥0.5 mg/dL) (*p*=0.001) (Tables 1 and 4). Zinc deficiency did not, however, correlate with number of CDI episodes (*p*=0.096) or number of CDI-related hospitalizations (*p*=0.733) (Table 1).

## 4. Discussion

Despite identification of CDI, procedural, and patient characteristics predisposing to FMT failure, recurrence of CDI after FMT remains difficult to fully predict. This suggests that other variables contributing to FMT outcomes have yet to be discovered. Zinc deficiency is a risk factor for diarrheal illness in certain clinical settings [15], and this study is the first to evaluate the relationship between zinc and recurrence of CDI following FMT.

In this cohort of patients receiving FMT for recurrent CDI, nearly 40% were zinc deficient based on evaluation of serum zinc. To the best of our knowledge, this is the first documentation of the rate of low zinc in patients with history of CDI. When adjusting for potential confounding variables, zinc deficiency was associated with increased CDI recurrence within 90 days after FMT. Among low zinc patients, receipt of zinc supplementation was associated with decreased CDI recurrence after FMT when analyzed in isolation and when controlling for risk factors for FMT failure.

The rate of low zinc in the study population was substantially higher than the estimated domestic prevalence of zinc deficiency of 4–7% [13]. Patients with history of recurrent CDI are likely predisposed to zinc depletion by the diarrhea and anorexia (leading to decreased dietary zinc intake) that characterize CDI episodes. Additionally, up to 10% of patients may develop post-infectious diarrhea-predominant irritable bowel syndrome following CDI episodes [22].

Zinc may play a physiologic role in preventing recurrence of CDI after FMT. In our study, low zinc was associated with increased recurrence when accounting for the known risk factors for FMT failure. Furthermore, zinc supplementation was associated with decreased recurrence risk. Studies suggest that zinc exerts antidiarrheal effects by inducing improved water and electrolyte absorption [23], mucosal integrity [24, 25], brush border enzymatic activity [26], and immunity [27]. Additionally, several recent animal-based studies suggest that zinc may facilitate the maintenance of a diverse microbiome [28–30]. It is possible that zinc aids in the prevention of post-FMT CDI through a combination of these effects. Given the high prevalence of low zinc observed in our study population and our findings suggesting that zinc deficiency may predispose patients to CDI recurrence, routine evaluation of serum zinc may be justified in patients with recurrent CDI to identify patients with zinc deficiency who may benefit from zinc supplementation.

Regardless of whether zinc deficiency predisposes patients to CDI, in our study, low zinc levels may serve simply as a marker of intestinal inflammation and dysbiosis, factors that could potentially increase the risk of FMT failure independent of total body zinc levels. Serum zinc is an acute phase reactant that decreases during the acute phase response [31]. As such, low serum zinc levels in patients with history of recurrent CDI likely reflect both zinc depletion and residual intestinal inflammation from prior CDI episodes. Evidence for zinc as a marker of inflammation in our population includes the correlation of low zinc with elevated CRP and low albumin. These correlations may be explained by the increased prevalence of IBD in the low zinc cohort.

Our study had several limitations. One major weakness is the small size of the study cohort, especially relative to the number of analyzed variables. The “low zinc-not supplemented” subcohort was particularly small, weakening conclusions regarding the impact of zinc supplementation. Additionally, as zinc is an acute phase reactant, measurements of serum zinc in patients with underlying inflammation may have been falsely depressed and not an accurate reflection of total body zinc. The retrospective nature of our study increases its susceptibility to documentation errors and limits our ability to account for important but previously undocumented variables. Most notably, we could not analyze the impact of post-FMT antibiotic exposure, as many patients received post-FMT care outside of our institution. Another limitation is the heterogeneity of the patient population with respect to history of IBD and zinc supplementation regimens. We refrained from excluding patients with history of IBD in order to maximize the size of the already limited study cohort. We felt that the inclusion of IBD patients was acceptable as the rate of CDI recurrence was not statistically significantly different between patients with and without IBD and as we controlled for IBD in our primary analysis using Cox regression. Given these inherent limitations, further study is required to definitively determine whether zinc deficiency increases incidence of CDI.

## 5. Conclusions

In conclusion, this is the first study to investigate the relationship between zinc deficiency and FMT outcomes. Zinc deficiency was present in 39% of patients receiving FMT for recurrent CDI at our institution. We found that low zinc levels were associated with higher rates of CDI recurrence after FMT when controlling for risk factors for FMT failure, and that zinc supplementation in the setting of zinc deficiency was associated with a reduced recurrence rate. While our small study has inherent limitations and should not alter the current management of CDI, the presented findings justify a prospective trial to determine the role of routine zinc measurement and repletion in the setting of CDI.

## Figures and Tables

**Table 1:** Study cohort characteristics.

	Normal zinc (*n* = 49)	Low zinc (*n* = 31)	Total (*n* = 80)	*p* value	Test utilized
*Zinc*					
Serum zinc (*µ*g/mL) (SD)	0.77 (0.09)	0.52 (0.08)	0.67 (0.15)	<0.001	*t*-test
*Patient characteristics*					
Age, mean (SD)	65.10 (15.98)	68.39 (17.94)	66.38 (16.73)	0.396	*t*-test
Female gender, *n* (%)	43 (87.76)	16 (51.61)	59 (73.75)	<0.001	Chi-square
BMI, mean (SD)	27.22 (6.56)	27.17 (8.37)	27.20 (7.29)	0.976	*t*-test
Immunocompromised state, *n* (%)	4 (8.16)	8 (25.81)	12 (15.00)	0.051	Fischer's exact
Charlson Comorbidity Index, mean (SD)	1.33 (1.83)	2.10 (2.20)	1.63 (2.00)	0.094	*t*-test
IBD, *n* (%)	2 (4.08)	10 (32.26)	12 (15.00)	<0.001	Fischer's exact
Ulcerative colitis, *n*	1	4	5		
Crohn's colitis, *n*	1	2	3		
Indeterminate colitis, *n*	0	4	4		
*CDI history*					
# CDIs, mean (SD)	3.59 (1.02)	4.10 (1.45)	3.79 (1.22)	0.096	*t*-test
# CDIs 6 mo before FMT, mean (SD)	2.84 (1.09)	2.74 (1.09)	2.80 (1.08)	0.706	*t*-test
# CDI hospitalizations, mean (SD)	1.12 (1.18)	1.23 (1.50)	1.16 (1.31)	0.733	*t*-test
# CDI hospitalizations 6 mo before FMT, mean (SD)	0.84 (1.11)	0.84 (1.04)	0.84 (1.07)	0.994	*t*-test

SD, standard deviation; BMI, body mass index; CDI, *Clostridium difficile* infection; IBD, inflammatory bowel disease; FMT, fecal microbiota transplant; mo, month.

**Table 2:** FMT outcomes.

	CDI recurrence within 90 days after FMT, *n* (%)	Days until CDI recurrence, median (range)
All subjects		
Normal zinc (*n* = 49)	3 (6.12)	72 (6–88)
Low zinc (*n* = 31)	5 (16.13)	15 (4–81)
Total (*n* = 80)	8 (10.00)	28 (4–88)
*p* value	0.097	0.456
Test utilized	Wilcoxon test	Mann–Whitney U
Low zinc subjects		
Not supplemented (*n* = 6)	3 (50.00)	14 (4–81)
Supplemented (*n* = 25)	2 (8.00)	28 (15–41)
Total (*n* = 31)	5 (16.13)	15 (4–81)
*p* value	0.014	0.564
Test utilized	Wilcoxon test	Mann–Whitney *U*

CDI, *Clostridium difficile* infection.

**Table 3:** Low zinc cohort characteristics.

	Not supplemented (*n* = 6)	Supplemented (*n* = 25)	Total (*n* = 31)	*p* value	Test utilized
*Zinc*					
Serum zinc (mcg/ml) (SD)	0.53 (0.08)	0.52 (0.08)	0.52 (0.08)	0.907	*t*-test
*Patient characteristics*					
Age, mean (SD)	75.33 (12.03)	66.72 (18.90)	68.39 (17.94)	0.299	*t*-test
Female gender, *n* (%)	4 (66.67)	12 (48.00)	16 (51.61)	0.654	Fischer's exact
BMI, mean (SD)	24.62 (5.34)	27.78 (8.93)	27.17 (8.37)	0.417	*t*-test
Immunocompromised state, *n* (%)	1 (16.67)	7 (28.00)	8 (25.81)	1.000	
Charlson Comorbidity Index, mean (SD)	2.00 (2.68)	2.12 (2.13)	2.10 (2.20)	0.907	
IBD, *n* (%)	1 (16.67)	9 (36.00)	10 (32.26)	0.634	Fischer's exact
Ulcerative colitis, *n*	1	3	4		
Crohn's colitis, *n*	0	2	2		
Indeterminate colitis, *n*	0	4	4		
*CDI history*					
# CDIs, mean (SD)	4.83 (1.47)	3.92 (1.41)	4.10 (1.45)	0.168	*t*-test
# CDIs 6 mo before FMT, mean (SD)	3.17 (1.17)	2.64 (1.08)	2.74 (1.09)	0.298	*t*-test
# CDI-hospitalizations, mean (SD)	2.33 (2.42)	0.96 (1.10)	1.23 (1.50)	0.228	*t*-test
# CDI-hospitalizations 6 mo before FMT, mean (SD)	1.50 (1.64)	0.68 (0.80)	0.84 (1.04)	0.283	*t*-test

SD, standard deviation; BMI, body mass index; CDI, *Clostridium difficile* infection; IBD, inflammatory bowel disease; FMT, fecal microbiota transplant; mo, month.

**Table 4:** Serum corollaries with low zinc.

	Normal zinc	Low zinc	Total	*p* value	Test utilized
*Albumin*	*n* = 39	*n* = 25	*n* = 64	—	
Mean (g/dl) (SD)	4.10 (0.30)	3.52 (0.41)	3.87 (0.45)	<0.001	*t*-test
Low (<3.5 g/dL), *n* (%)	0 (0.00)	7 (28.00)	7 (10.94)	0.001	Fischer's exact
*C-reactive protein*	*n* = 36	*n* = 28	*n* = 64	—	
Mean (mg/dL) (SD)	0.68 (0.91)	1.94 (2.10)	1.23 (1.66)	0.006	*t*-test
High (≥0.5 mg/dL), *n* (%)	15 (41.67)	23 (82.14)	38 (59.38)	0.001	Chi-square
*White blood cell count*	*n* = 35	*n* = 26	*n* = 61	—	
Mean (k cells/mm^3^) (SD)	8.03 (2.19)	7.24 (2.33)	7.91 (2.72)	0.179	*t*-test
High (>11,000 cells/mm^3^), *n* (%)	3 (8.57)	0 (0.00)	3 (4.92)	0.254	Fischer's exact

SD, standard deviation. The number of patients listed per laboratory value varies as some patients did not have albumin, C-reactive protein, and/or white blood cell count recorded at time of zinc measurement.

## Data Availability

The data used to support the findings of this study are included within the supplementary information file entitled “S1 Data.”

## Supplementary Materials

Supplementary Materials Table S1: Cox regression output for full cohort. Table S2: Cox regression output for low zinc cohort.
