# Supplementary material for: Zinc Deficiency and the Recurrence of Clostridium difficile Infection after Fecal Microbiota Transplant: A Retrospective Cohort Study
Source: J Nutr Metab. 2018 Oct 10;2018:9682975. doi: 10.1155/2018/9682975 (PMC6199870; doi:10.1155/2018/9682975)
Supplement: Supplementary Materials — Table S1: Cox regression output for full cohort. Table S2: Cox regression output for low zinc cohort. [file 9682975.f1.zip › Revised Supplementary Tables.docx]

**Supplementary Tables**

**S1 Table 1: Cox Regression Output for Full Cohort**

| **Variable** | **Hazard Ratio** | **95% Hazard Ratio Confidence Interval** | **p-value** | **Test**  **Utilized** |
| --- | --- | --- | --- | --- |
| Low Zinc | 11.327 | 2.162-59.336 | **0.004** | Cox regression |
| Zinc Supplementation | 0.119 | 0.019-0.738 | **0.022** | Cox regression |
| Charlson Comorbidity Index | 1.441 | 1.087-1.911 | **0.011** | Cox regression |
| CDI Hospitalizations | - | - | 0.659 | Cox regression |
| Age | - | - | 0.527 | Cox regression |
| Immunocompromised Status | - | - | 0.553 | Cox regression |
| Gender | - | - | 0.313 | Cox regression |
| IBD | - | - | 0.273 | Cox regression |

Note: Variables lacking associated hazard ratios and 95% confidence intervals were removed from the Cox regression model via backward selection using p<0.1 as the staying criterion.

**S1 Table 2: Cox Regression Output for Low Zinc Cohort**

| **Variable** | **Hazard Ratio** | **95% Hazard Ratio Confidence Interval** | **p-value** | **Test**  **Utilized** |
| --- | --- | --- | --- | --- |
| Zinc Supplementation | 0.102 | 0.015-0.704 | **0.021** | Cox regression |
| Charlson Comorbidity Index | 1.521 | 1.015-2.279 | **0.011** | Cox regression |
| Immunocompromised Status | - | - | 1.000 | Cox regression |
| Gender | - | - | 1.000 | Cox regression |
| CDI Hospitalizations | - | - | 0.536 | Cox regression |
| Age | - | - | 0.423 | Cox regression |
| IBD | - | - | 0.218 | Cox regression |

Note: Variables lacking associated hazard ratios and 95% confidence intervals were removed from the Cox regression model via backward selection using p<0.1 as the staying criterion.
